# Comparison of CHOP‐19 and CHOP‐25 for treatment of peripheral nodal B‐cell lymphoma in dogs: A European multicenter retrospective cohort study

**DOI:** 10.1111/jvim.17222

**Published:** 2024-10-18

**Authors:** Charles Hawkes, Joanna Morris, Spela Bavcar, Craig Wilkie, Surajit Ray, Charlotte Auquier, Sarah Benjamin, Juan Borrego Massó, Sébastien Bottin, Owen Davies, Isabelle Desmas‐Bazelle, Anat Einhorn, Celia Figueroa‐Gonzalez, Katerina Holenova, Elisavet Kritsotalaki, Kerry Peak, Katherine Smallwood, Elisabetta Treggiari, Paola Valenti, Miguel Garcia de la Virgen, Quentin Fournier

**Affiliations:** ^1^ The Royal (Dick) School of Veterinary Studies University of Edinburgh Roslin United Kingdom; ^2^ University of Glasgow Glasgow United Kingdom; ^3^ Small Animal Specialist Hospital North Ryde New South Wales Australia; ^4^ University of Liege Liege Belgium; ^5^ Davies Veterinary Specialists Hitchin United Kingdom; ^6^ Hospital Aúna Especialidades Veterinarias IVC‐Evidensia Valencia Spain; ^7^ Aura Veterinary Guildford United Kingdom; ^8^ Bristol Vet Specialists Bristol United Kingdom; ^9^ Royal Veterinary College London United Kingdom; ^10^ Koret School of Veterinary Medicine Rehovot Israel; ^11^ Wear Referrals Bradbury United Kingdom; ^12^ Pride Veterinary Referrals Derby United Kingdom; ^13^ Southfields Veterinary Specialists Basildon United Kingdom; ^14^ Anderson Moores Winchester United Kingdom; ^15^ North Downs Specialists Referrals Bletchingley United Kingdom; ^16^ Oncopets Milan Italy; ^17^ Clinica Veterinaria Malpensa AniCura Milan Italy

**Keywords:** canine, CHOP‐19, CHOP‐25, lymphoma, progression‐free survival

## Abstract

**Background:**

Peripheral nodal B‐cell lymphomas (PNBCL) represent the most common presentation of lymphomas in dogs. Multiagent CHOP (C = cyclophosphamide, H = hydroxydaunorubicin [Doxorubicin], O = Oncovin, P = prednisolone)‐based chemotherapy protocols have been widely accepted as gold standard 1st‐line treatment. CHOP‐25 and CHOP‐19 are most commonly prescribed but have never been directly compared.

**Objectives:**

Our primary aim was to compare outcomes of dogs diagnosed with PNBCL, treated using a 1st‐line CHOP‐19 or CHOP‐25 protocol. A secondary objective was to determine the impact of protocol‐related variables on outcomes.

**Animals:**

Five hundred two dogs from 16 European oncology referral centers. One hundred fifty‐five dogs were treated with CHOP‐19 and 347 dogs with CHOP‐25.

**Methods:**

Retrospective, multicentric cohort study of dogs diagnosed with PNBCL between 2014 and 2021.

**Results:**

The 6‐month, 1‐year, and median progression‐free survival (PFS) were 56.5% (95% confidence interval [CI], 49.2‐65.0), 14.1% (95% CI, 9.4‐21.0), and 196 days (95% CI, 176‐233) with CHOP‐19; and 56.4% (95% CI, 51.4‐61.9), 17% (95% CI, 13.4‐21.6), and 209 days (95% CI, 187‐224) with CHOP‐25. The 1‐year, 2‐year and median overall survival (OS) were 36.9% (95% CI, 29.7‐46.0), 13.5% (95% CI, 8.6‐21.1), and 302 days (95% CI, 249‐338) with CHOP‐19; and 42.8% (95% CI, 37.7‐48.7), 15.4% (95% CI, 11.7‐20.4), and 321 days (95% CI, 293‐357) with CHOP‐25. No significant difference in PFS and OS was found between the 2 protocols.

**Conclusions and Clinical Importance:**

Our study confirmed similar outcomes for dogs with PNBCL treated with 1st‐line CHOP‐19 or CHOP‐25. Both protocols therefore could be used as a standard of care in future trials.

**Abbreviations**
ACVIM: American College of Veterinary Internal Medicine
AE: adverse event
CI: confidence interval
CR: complete response
CT: computed tomography
DD: dose delay
DI: dose intensity
DLBCL: diffuse large B‐cell lymphoma
DR: dose reduction
EBVS: European Board of Veterinary Specialization
ECVIM: European College of Veterinary Internal Medicine
HR: hazard ratio
MST: median survival time
ORR: objective response rate
OS: overall survival
PARR: PCR for antigen receptor rearrangement
PFS: progression‐free survival
PNBCL: peripheral nodal B‐cell lymphoma
PR: partial response
RDI: relative dose intensity
VCOG: Veterinary Comparative Oncology Group
WHO: World Health Organization

## 1 INTRODUCTION

Lymphoma is the most common hematopoietic malignancy diagnosed in dogs.^1^, ^2^ Approximately 80% of lymphomas in dogs present in multicentric form, typically with generalized peripheral lymphadenopathy.^3^ Among these, approximately 70% to 80% display B‐cell immunophenotype.^4^, ^5^ Intermediate to high grade B‐cell lymphomas represent approximately 47% of all lymphomas in dogs,^6^ with diffuse large B‐cell lymphomas (DLBCL) being the most common subtype, representing approximately 40% of lymphomas.^7^ Most B‐cell lymphomas in dogs exhibit aggressive behavior, whereas a minority progress slowly, exhibiting indolent behavior.^6^ Peripheral nodal B‐cell lymphomas (PNBCL) mainly include DLBCL, but other subtypes such as Burkitt‐like lymphomas,^8^ and marginal zone lymphomas^9^ also are represented. One of the most commonly used chemotherapy protocols for multicentric lymphoma, CHOP (C = cyclophosphamide, H = hydroxydaunorubicin [Doxorubicin], O = Oncovin, P = prednisolone)‐25, was proposed over 20 years ago.^10^ CHOP‐19 was subsequently suggested as a condensed version.^11^ There is currently no consensus on 1st‐line treatment of lymphomas in dogs, but a recent nonpublished survey confirmed these 2 protocols are the most commonly prescribed by European oncology specialists. Shorter protocols (CHOP‐15, CHOP‐12) have been described,^4^, ^12^, ^13^, ^14^ but have not gained popularity in Europe.

For the last 20 years, chemotherapy protocols used to treat PNBCL in dogs have not changed substantially. It is assumed CHOP‐19 and CHOP‐25 are associated with similar outcomes, but these protocols have never been compared in a randomized clinical trial. Median progression‐free survival (PFS) and median survival times (MST) of dogs with DLBCL treated with CHOP‐19 are 188 to 233 days and 245‐325 days, respectively.^15^, ^16^, ^17^ Median PFS and MST of dogs with DLBCL treated with CHOP‐25 are 252 days and 341 days.^18^ These findings remain difficult to compare. More recently, CHOP‐19 was associated with a better outcome compared with CHOP‐12.^14^ Possible explanations included increased vincristine dose intensity (DI) and overall protocol duration.^14^ The 1st 2 cycles of CHOP‐19 and CHOP‐25 are identical, but CHOP‐19 maintains higher DI in the protocol's 2nd half, whereas CHOP‐25 has a longer duration. It is possible the protocols are not associated with identical outcomes, which also could vary based on the dog's initial response.^19^

One advantage of CHOP‐19 is that the shorter protocol maximizes time away from treatment, whereas CHOP‐25 distributes the financial and logistical burden in the latter 2 cycles by using visits every 2 weeks.

Our primary aim was to compare outcomes of dogs diagnosed with PNBCL and treated with 1st‐line CHOP‐19 or CHOP‐25 protocols and help define a gold standard 1st‐line treatment, which could be used in European guidelines and as a control group in future trials. Our primary objective was to compare 6‐month PFS between the 2 protocols, and we hypothesized that CHOP‐19 would be associated with a better outcome, because of an overall increased DI. A secondary objective was to determine the impact of protocol‐related variables (protocol modifications, adverse events [AE], response to treatment) on outcomes.

## 2 MATERIALS AND METHODS

### 2.1 Study setting

An initial survey was sent electronically to 38 European veterinary referral centers, each with at least 1 European College of Veterinary Internal Medicine (ECVIM) or American College of Veterinary Internal Medicine (ACVIM) boarded–certified oncologist, or both, identified from the European Board of Veterinary Specialization (EBVS; https://ebvs.eu/) and ACVIM (https://www.acvim.org/) websites. The goal was to confirm the most prescribed 1st‐line chemotherapy protocols for PNBCL, identify potential study centers, and estimate the number of cases available. From the 21 replies, CHOP‐19 was prescribed at 10 centers, CHOP‐25 at 7 centers, and other protocols at 7 centers. It was predicted from the survey that the number of available cases would be sufficient to proceed with the study.

Sixteen European academic (n = 5) and private (n = 11) centers, routinely recording treatment response using Veterinary Cooperative Oncology Group (VCOG) response evaluation criteria for peripheral nodal lymphoma in dogs^19^ and recording AEs using VCOG‐Common Terminology Criteria for Adverse Events (VCOG‐CTCAE) 1.1,^20^ were able to participate. United Kingdom, Italy, Spain, Belgium and Israel were represented. Nine centers prescribed CHOP‐19, and 12 centers CHOP‐25.

Dogs were recruited during the 7‐year period from June 2014 to June 2021, and data collection was closed in June 2022 to allow a minimum 1‐year follow‐up. Ethical approval for our study was obtained by the Veterinary Ethic Review Committee of the University of Edinburgh (reference 110.21).

### 2.2 Dog selection

Dogs were eligible for inclusion if they had (1) a cytological or histological diagnosis of lymphoma made by a clinical or anatomical pathologist, respectively; (2) a peripheral nodal form, where the lymph nodes contain most of the tumor burden; (3) a B‐cell immunophenotype confirmed using either flow cytometry, immunohistochemistry, immunocytochemistry or PCR for antigen receptor rearrangement (PARR); and (4) World Health Organization (WHO) Stage 3 or above,^21^ treated with 1st‐line CHOP‐19 or CHOP‐25. Dogs were excluded if they had (1) a confirmed or suspected low‐grade or clinically indolent lymphoma; (2) a suspected extranodal form of lymphoma; (3) severe preexisting comorbidities; (4) confirmed *ABCB1* gene mutation; (5) received prior treatment with corticosteroids for >14 days. Severe preexisting comorbidities consisted of conditions with reasonable likelihood of causing death within 12 months (eg, other advanced cancer, cardiac failure, renal failure).

### 2.3 Interventions

Dogs diagnosed with PNBCL and treated with 1st‐line CHOP‐19 or CHOP‐25 were identified, as reported in Table 1.^10^, ^14^ Initiation and termination dates of protocols were recorded. Switching doxorubicin to another anthracycline (eg, epirubicin, mitoxantrone), cyclophosphamide to chlorambucil, and vincristine to vinblastine, because of documented AEs or to prevent the development of toxicity in high‐risk individuals was accepted. Induction with L‐asparaginase (L‐CHOP) also was accepted.

**TABLE 1:** Chemotherapy protocols compared in the study.

CHOP‐19	Week															
	1	2	3	4	6	7	8	9	11	12	13	14	16	17	18	19
Vincristine 0.7 mg/m^2^ IV	X		X		X		X		X		X		X		X	
Cyclophosphamide 250 mg/m^2^ PO		X				X				X				X		
Doxorubicin^a^ 30 mg/m^2^ IV				X				X				X				X
Prednisolone^b^ PO	X	X	X	X												

CHOP‐25	Week															
	1	2	3	4	6	7	8	9	11	13	15	17	19	21	23	25
Vincristine 0.7 mg/m^2^ IV	X		X		X		X		X		X		X		X	
Cyclophosphamide 250 mg/m^2^ PO		X				X				X				X		
Doxorubicin^a^ 30 mg/m^2^ IV				X				X				X				X
Prednisolone^b^ PO	X	X	X	X												

^a^ Dogs <15 kg receive doxorubicin at 25 mg/m^2^.

^b^ 2 mg/kg q24h on week 1, 1.5 mg/kg q24h on week 2, 1 mg/kg q24h on week 3, 0.5 mg/kg q24h on week 4.

Abbreviation: CHOP, C = cyclophosphamide, H = hydroxydaunorubicin (Doxorubicin), O = Oncovin, P = prednisolone.

### 2.4 Data collection

Information obtained on dogs included sex, age at diagnosis, breed, weight, method of diagnosis and immunophenotyping, grade and subtype of lymphoma if histology was performed, stage, substage, presence of so‐called B‐symptoms^16^ and clinical signs.

Baseline CBC and serum biochemistry results were recorded for each dog and abnormalities were identified using the reference ranges provided by the performing laboratory. Chemotherapy AEs were graded using VCOG‐CTCAE 1.1^20^ criteria. Hematology tests performed postchemotherapy identified the frequency and grade of neutropenia. Nonhematological AEs, such as gastrointestinal, were recorded based on owner accounts and observations of the attending clinician. Clinical stage and substage were recorded using the WHO clinical staging system for lymphoma in dogs.^21^ Abdominal and thoracic imaging, liver and spleen cytology and bone marrow aspirate results all were recorded. Hospitalization was defined as dogs requiring IV treatment on a day or overnight basis.

Response to treatment was recorded based on VCOG standardized criteria.^19^ Complete response (CR) was defined as the lymph nodes decreasing to normal size and no evidence of disease elsewhere. Partial response (PR) was defined as a 30% reduction in the size of the target lymph nodes. Progressive disease (PD) was defined as the increase in target lymph node size of at least 20% beyond the smallest recorded measurement or development of new lesions. Stable disease (SD) was defined as either a <30% decrease or 20% increase, or no change in lymph node size, without development of new lesions.^19^ Details of rescue protocol were documented after confirmation of disease progression.

### 2.5 Data analysis

Categorical variables were described using frequencies, and 95% CI when appropriate, whereas continuous variables were described using median and range. The distribution of dog characteristics, protocol alterations and AEs between the 2 protocols were compared using Pearson's Chi‐squared test with Yates continuity correction for categorical variables, Mann‐Whitney test for continuous variables, and Poisson regressions for count variables.

Kaplan‐Meier estimation was used to determine dogs' PFS and overall survival (OS) probabilities, stratified by individual categorical variables. The Log‐Rank test was used to determine the differences between survival curves, and Z‐tests for differences in proportions were used to determine the differences in PFS and OS at specific timepoints. The Cox proportional hazards method was used to model the relationship between PFS or OS and continuous explanatory variables, and to model the relationship between PFS or OS and categorical variables accounting for other continuous and categorical explanatory variables. Hazard ratios (HR) for cancer progression were determined using the Cox proportional hazards method.

Progression‐free survival was defined as the time from initiation of either CHOP protocol to disease progression or death. Overall survival was defined as the time from initiation of CHOP protocol to death from any cause. Objective response rate (ORR) was defined as dogs that achieved CR or PR. Dogs were censored in the PFS analysis if they did not have progression documented at the time of last follow‐up or were lost to follow‐up. Dogs were censored in the survival analysis if they were alive at the last follow‐up or lost to follow‐up.

The DI of vincristine, cyclophosphamide and doxorubicin was calculated as described previously.^22^ The standard planned total administration dose for each chemotherapy drug was calculated and divided by the total number of weeks of the protocol. Doxorubicin DI was determined for dogs ≥15 kg using 30 mg/m^2^ dosage and for dogs <15 kg, using 25 mg/m^2^ dosage, even if 1 mg/kg also was considered a standard dosage by some clinicians, to allow relative DI (RDI) calculation across the cohort. Delivered DI for each chemotherapy drug was calculated using the formula: sum of all doses received (mg/m^2^)/(total days of treatment/7). Relative DI was calculated dividing the delivered DI by the planned DI of the protocol. Because RDI can be higher when the protocol is discontinued, it also was determined specifically for completed protocols. Dose intensity was not calculated if a change in chemotherapy drug occurred during the protocol. Calculations of DI were made using Microsoft Excel software, version 2404.

Maximal gastrointestinal toxicity grade was recorded for each chemotherapy drug during the protocol. The overall number, and the number of grade 1, 2, 3, 4, and 5 neutropenia toxicities, were recorded for each chemotherapy drug. A neutropenia score for each chemotherapy drug was calculated as follows: number of grade 1 neutropenias + 2 X (number of grade 2 neutropenias) + 3 X (number of grade 3 neutropenias) + 4 X (number of grade 4 neutropenias). A neutropenia score for the overall protocol was calculated as the sum of each individual chemotherapy drug neutropenia score. A maximal gastrointestinal toxicity score for the overall protocol also was calculated as the sum of each individual chemotherapy drug maximal gastrointestinal toxicity grade.

Analyses were performed using R software, version 4.4.0, with packages ggplot2 version 3.5.1,^23^ survival version 3.5‐8^24^ and survminer version 0.4.9.^25^

## 3 RESULTS

### 3.1 Dogs

Five hundred four dogs fulfilled the criteria to be included in the study. One hundred fifty‐five dogs from 9 centers were treated with CHOP‐19 and 349 dogs from 12 centers were treated with CHOP‐25. Four dogs originally intended to receive CHOP‐19 instead received CHOP‐25 because of toxicity in the initial cycles. Data for 2 dogs that received CHOP‐25 were removed from the study, because of inconsistencies in the data. Individual characteristics are compared in Table 2. No significant differences were found for sex, body weight, age, breed distribution, minimum stage, substage, presence of B‐symptoms, pretreatment with corticosteroids for <14 days before commencing chemotherapy, presence of anemia, presence of hypercalcemia, and extranodal involvement between the CHOP‐19 and CHOP‐25 groups. Dogs receiving CHOP‐25 were more frequently treated in private hospitals (*P* = .002), and in non‐UK countries (*P* = .01), compared with dogs receiving CHOP‐19. Treatment in an academic hospital was not associated with any significant difference in PFS (*P* = .07), 6‐month PFS (*P* = .93), OS (*P* = .94), 1‐year OS (*P* = .31), and 2‐year OS (*P* = .57). Treatment in the UK was not associated with any significant difference in PFS (*P* = .87), 6‐month PFS (*P* = .74), OS (*P* = .8), 1‐year OS (*P* = .87), and 2‐year OS (*P* = .78).

**TABLE 2:** Baseline dog demographic and clinical characteristics.

Variable	CHOP‐19 (155 dogs)	CHOP‐25 (347 dogs)	*P*‐value
Sex			.14
Female entire	6 (3.9%)	28 (8.1%)	
Female neutered	62 (40%)	139 (40.1%)	
Male entire	22 (14.2%)	62 (17.9%)	
Male neutered	65 (41.9%)	118 (34%)	
Body weight (kg)			.98
Median (range)	22.5 (3.6‐66)	22.7 (2.1‐86)	
Age (years)			.72
Median (range)	8 (1.1‐16)	8.3 (1.5‐15.4)	
Breed distribution			.81
Crossbreed	39 (25.2%)	87 (25.1%)	
Border Collie	5 (3.2%)	25 (7.2%)	
Cocker spaniel	6 (3.9%)	14 (4%)	
German shepherd	6 (3.9%)	12 (3.5%)	
Golden retriever	11 (7.1%)	18 (5.2%)	
Jack Russell terrier	5 (3.2%)	11 (3.2%)	
Labrador retriever	8 (5.2%)	20 (5.8%)	
Other	75 (48.4%)	160 (46.1%)	
Minimum stage			.75
3	78 (50.3%)	182 (52.4%)	
4	43 (27.7%)	99 (28.5%)	
5	34 (21.9%)	66 (19%)	
Extranodal involvement			.61
No	133 (85.8%)	305 (87.9%)	
Yes	22 (14.2%)	42 (12.1%)	
Substage			.12
a	97 (62.6%)	190 (54.8%)	
b	58 (37.4%)	157 (45.2%)	
Presence of B‐symptoms			.43
No	101 (65.2%)	240 (69.2%)	
Yes	54 (34.8%)	107 (30.8%)	
Pretreatment with corticosteroids (<14 days)			.99
No	133 (85.8%)	296 (85.3%)	
Yes	22 (14.2%)	51 (14.7%)	
Presence of anemia			.9
No	103 (71%)	241 (72.2%)	
Yes	42 (29%)	93 (27.8%)	
Presence of hypercalcemia			.25
No	135 (97.1%)	317 (99.1%)	
Yes	4 (2.9%)	3 (0.9%)	
Institution			.002*
Academic	63 (40.6%)	92 (26.5%)	
Private	92 (59.4%)	255 (73.5%)	
Country			.01*
Non‐UK	34 (21.9%)	117 (33.7%)	
UK	121 (78.1%)	230 (66.3%)	

* Significant differences (*P* < .05).

Abbreviation: CHOP, C = cyclophosphamide, H = hydroxydaunorubicin (Doxorubicin), O = Oncovin, P = prednisolone.

A morphological diagnosis of lymphoma was obtained by cytology in 431/502 (85.9%) dogs, and by histopathology in 71/502 (14.1%) dogs. Immunophenotyping was performed using flow cytometry in 249/502 (49.6%) dogs, immunocytochemistry in 99/502 (19.7%) dogs, PARR in 97/502 (19.3%) dogs, and immunohistochemistry in 71/502 (14.1%) dogs.

Baseline blood tests including CBC and serum biochemistry were performed in all dogs, and 377/502 (75.1%) had blood smear evaluation. Thoracic imaging was performed in 271/502 (54%) dogs, using thoracic radiography in 235/502 (46.8%) dogs and computed tomography (CT) in 36/502 (7.2%) dogs. Abdominal imaging was performed in 297/502 (59.2%) dogs, using ultrasonography in 269/502 (53.6%) dogs, CT in 27/502 (5.4%) dogs, and radiography in 1/502 (0.2%) dogs. Liver and spleen aspirates for cytology were obtained in 152/502 (30.3%) and 165/502 (32.9%) dogs, respectively. Bone marrow aspirates were obtained in 54/502 (10.8%) dogs.

### 3.2 Protocols

The main protocol modifications are summarized in Table 3. L‐asparaginase administration was not associated with any significant difference in PFS (*P* = .85) or OS (*P* = .77). The 1st doses of vincristine, cyclophosphamide and doxorubicin were decreased in 141/502 (28.1%) dogs, 47/501 (9.4%) dogs, and 104/488 (21.3%) dogs, respectively. Only the 1st cyclophosphamide dose was more frequently decreased in CHOP‐25 (*P* < .001). Most initial doxorubicin dose modifications were attributable to a 1 mg/kg dosage in <15 kg dogs, which was considered standard by some clinicians. None of these initial dose alterations were associated with any significant differences in PFS and OS (Table 3).

**TABLE 3:** Distribution of protocol modifications and impact on outcome.

	Distribution			PFS		OS	
Variable	CHOP‐19 (155 dogs)	CHOP‐25 (347 dogs)	*P*‐value	Hazard ratio	*P*‐value	Hazard ratio	*P*‐value
L‐asparaginase induction			.093	1.03	.846	1.04	.76
No	138 (89%)	287 (82.7%)					
Yes	17 (11%)	60 (17.3%)					
1st vincristine < 0.7 mg/m^2^			.279	1	.963	1.08	.49
No	117 (75.5%)	244 (70.3%)					
Yes	38 (24.5%)	103 (29.7%)					
1st cyclophosphamide <250 mg/m^2^			<.001*	0.96	.819	1.09	.61
No	151 (98.1%)	303 (87.3%)					
Yes	3 (1.9%)	44 (12.7%)					
1st doxorubicin < 30 mg/m^2^ (for dogs ≥ 15 kg)			.411	1.02	.917	1	1.0
No	88 (90.7%)	217 (86.8%)					
Yes	9 (9.3%)	33 (13.2%)					
1st doxorubicin <25 mg/m^2^ (for dogs <15 kg)			1	0.94	.704	0.97	.89
No	27 (56.2%)	52 (55.9%)					
Yes	21 (43.8%)	41 (44.1%)					
Vincristine RDI							
All dogs (median [range])	0.91 (0.45‐2.26)	0.92 (0.33‐4.26)	.117	7.04	<.001*	4.33	<.001*
Completed protocols (median [range])	0.88 (0.48‐1.06)	0.89 (0.33‐1.48)	.518	2.17	.128	2.09	.18
Cyclophosphamide RDI							
All dogs (median [range])	0.96 (0.6‐4.5)	0.9 (0.25‐5.86)	.112	2.56	<.001*	2.09	<.001*
Completed protocols (median [range])	0.93 (0.6‐1.11)	0.87 (0.44‐1.46)	.01*	5.64	<.001*	3.91	.01*
Doxorubicin RDI							
All dogs (median [range])	0.88 (0.38‐2.43)	0.93 (0.25‐2.16)	.014*	4.73	<.001*	2.32	.001*
Completed protocols (median [range])	0.91 (0.46‐1.17)	0.93 (0.3‐1.45)	.241	1.86	.176	1.44	.48
Number of dose delays			.005*	0.87	<.001*	0.9	.003*
Median (range)	0 (0‐6)	1 (0‐10)					
Number of dose reductions			.008*	0.9	<.001*	0.95	.03*
Median (range)	0 (0‐6)	1 (0‐12)					

Abbreviations: CHOP, C = cyclophosphamide, H = hydroxydaunorubicin (Doxorubicin), O = Oncovin, P = prednisolone; PFS, progression‐free survival; OS, overall survival; RDI, relative dose intensity.

* Significant differences (*P* < .05).

First‐line protocols were completed in 90/155 (58.1%) dogs prescribed CHOP‐19, and in 182/347 (52.4%) dogs prescribed CHOP‐25 (*P* = .28). Reasons for protocol discontinuation were PD (160/230, 69.6%), financial limitation (9/230, 3.9%), toxicity (20/230, 8.7%), and other reasons (41/230, 17.8%), with a similar distribution between CHOP‐19 and CHOP‐25 protocols (*P* = .29). Other reasons included, but were not limited to, coronavirus (COVID‐19) restrictions, owner's decision, congestive heart failure suspected to be unrelated to doxorubicin, intestinal perforation and intervertebral disc protrusion.

Vincristine was changed to vinblastine in 7/155 (4.5%) dogs receiving CHOP‐19 and in 13/347 (3.7%) dogs receiving CHOP‐25 (*P* = .87). Cyclophosphamide was changed to another alkylating agent in 13/155 (8.4%) CHOP‐19 dogs and in 32/347 (9.2%) CHOP‐25 dogs (*P* = .89). Doxorubicin was changed to another anthracycline in 9/155 (5.8%) CHOP‐19 dogs and in 18/347 (5.2%) CHOP‐25 dogs (*P* = .94).

In the CHOP‐19 group, 41 (26.5%) dogs received 1 dose reduction (DR), 18 (11.6%) dogs 2 DRs, 10 (6.5%) dogs 3 DRs and 1 (0.6%) dog each 4 and 6 DRs. In the CHOP‐25 group, 96 (27.7%) dogs received 1 DR, 39 (11.2%) dogs 2 DRs, 15 (4.3%) dogs 3 DRs, 11 (3.2%) dogs 4 DRs, 7 (2.0%) dogs 5 DRs, 5 (1.4%) dogs 6 DRs, 6 (1.7%) dogs 7 DRs, 2 (0.6%) dogs 8 DRs, 4 (1.2%) dogs 9 DRs, 3 (0.9%) dogs 10 DRs, 1 (0.3%) dog 11 DRs and 4 (1.2%) dogs 12 DRs. The number of DRs was significantly higher in dogs receiving CHOP‐25 compared with CHOP‐19 (*P* = .01), and it was positively associated with PFS and OS (Table 3).

In the CHOP‐19 group, 30 dogs had 1 dose delay (DD), 19 dogs 2 DDs, 10 dogs 3 DDs, and 2 dogs each had 4 and 6 DDs. In the CHOP‐25 group, 97 dogs had 1 DD, 45 dogs 2 DDs, 22 dogs 3 DDs, 14 dogs 4 DDs, 6 dogs 5 DDs, 1 dog 6 DDs, 3 dogs 7 DDs, 1 dog 9 DDs and 2 dogs 10 DDs. The number of DDs was significantly higher in dogs receiving CHOP‐25 compared with CHOP‐19 (*P* = .005), and it was positively associated with PFS and OS (Table 3).

Delivered chemotherapy DI (mg/m^2^/week) was significantly higher for CHOP‐19 compared with CHOP‐25 (*P* < .001 for all 3 drugs), with vincristine at 0.28 (0.14‐0.70) and 0.21 (0.08‐0.98), cyclophosphamide at 53.0 (33.1‐250) and 37.6 (10.5‐244), doxorubicin at 5.5 (2.1‐16.2) and 4.5 (1.2‐10.0), respectively (all reported as median [range]). Doxorubicin RDI was significantly higher for CHOP‐25 compared with CHOP‐19 (*P* = .01), and cyclophosphamide RDI was significantly higher for CHOP‐19 compared with CHOP‐25 for dogs that completed the protocol only (*P* = .01). Chemotherapy RDI was negatively associated with PFS and OS. When only completed protocols were analyzed, vincristine and doxorubicin RDI was no longer significantly associated with PFS and OS. Relative DI also was negatively correlated with the number of neutropenias (Figure S1).

### 3.3 Adverse events

Adverse events^20^ were reported in 135/155 (86.2%) dogs treated with CHOP‐19, and 317/347 (91.4%) dogs treated with CHOP‐25 (*P* = .19). The AE summary is presented in Table 4. Two grade 5 toxicities were recorded, 1 each postvincristine and postdoxorubicin, respectively. Grade 3/4 gastrointestinal toxicity was recorded in 44/502 (8.8%) dogs after vincristine administration, 26/502 (5.2%) dogs after cyclophosphamide administration, and 34/502 (6.8%) dogs after doxorubicin administration. Grade 3/4 neutropenia was recorded in 45/502 dogs (8.9%) after vincristine administration, 47/502 dogs (9.4%) after cyclophosphamide administration, and 15/502 dogs (2.9%) after doxorubicin administration.

**TABLE 4:** : Distribution of adverse events and impact on outcome.

Variable	Distribution			PFS		OS	
	CHOP‐19 (155 dogs)	CHOP‐25 (347 dogs)	*P*‐value	Hazard ratio	*P*‐value	Hazard ratio	*P*‐value
**Vincristine** (median [range])							
Maximal gastrointestinal toxicity grade	1 (0‐4)	1 (0‐5)	**<.001 (estimate = 0.359)** *	0.99	.79	0.95	.31
Number of neutropenia events	0 (0‐6)	0 (0‐8)	**<.001 (estimate = 0.521)** *	0.85	**<.001** *	0.86	**<.001** *
Neutropenia highest grade	0 (0‐4)	0 (0‐4)	**.01 (estimate = 0.346)** *	0.87	**.01** *	0.9	**.04** *
Neutropenia score	0 (0‐8)	0 (0‐12)	**<.001 (estimate = 0.384)** *	0.92	**.001** *	0.93	**.003** *
**Cyclophosphamide** (median [range])							
Maximal gastrointestinal toxicity grade	0 (0‐4)	0 (0‐3)	.21 (estimate = −0.177)	0.95	.36	1.01	.85
Number of neutropenia events	0 (0‐4)	0 (0‐3)	.38 (estimate = −0.124)	0.75	**<.001** *	0.76	**<.001** *
Neutropenia highest grade	0 (0‐4)	0 (0‐4)	.05 (estimate = −0.241)	0.85	**<.001** *	0.83	**<.001** *
Neutropenia score	0 (0‐13)	0 (0‐9)	**<.001 (estimate = −0.394)** *	0.88	**<.001** *	0.88	**<.001** *
**Doxorubicin** [median (range)]							
Maximal gastrointestinal toxicity grade	0 (0‐4)	0 (0‐5)	.25 (estimate = 0.14)	0.98	.67	1.02	.7
Number of neutropenia events	0 (0‐4)	0 (0‐3)	.25 (estimate = 0.248)	0.79	**.01** *	0.83	**.03** *
Neutropenia highest grade	0 (0‐4)	0 (0‐4)	.47 (estimate = 0.138)	0.84	**.01** *	0.86	**.03** *
Neutropenia score	0 (0‐12)	0 (0‐8)	.84 (estimate = −0.033)	0.89	**0.012** *	0.91	.06
**Chemotherapy protocol** (median [range])							
Sum maximal gastrointestinal toxicity grade	2 (0‐10)	2 (0‐8)	**.01 (estimate = 0.176)** *	0.97	.27	0.98	.43
Number of neutropenia events	1 (0‐10)	1 (0‐13)	**.003 (estimate = 0.262)** *	0.87	**<.001** *	0.87	**<.001** *
Neutropenia score	1 (0‐18)	1 (0‐21)	.69 (estimate = 0.026)	0.92	**<.001** *	0.93	**<.001** *
**Dogs hospitalized**			.05	1.41	**.003** *	1.44	**.003** *
No	131 (84.5%)	265 (76.4%)					
Yes	24 (15.5%)	82 (23.6%)					

Abbreviations: CHOP, C = cyclophosphamide, H = hydroxydaunorubicin (Doxorubicin), O = Oncovin, P = prednisolone; PFS, progression‐free survival; OS, overall survival.

* Significant differences (*P* < .05).

**FIGURE 1:**
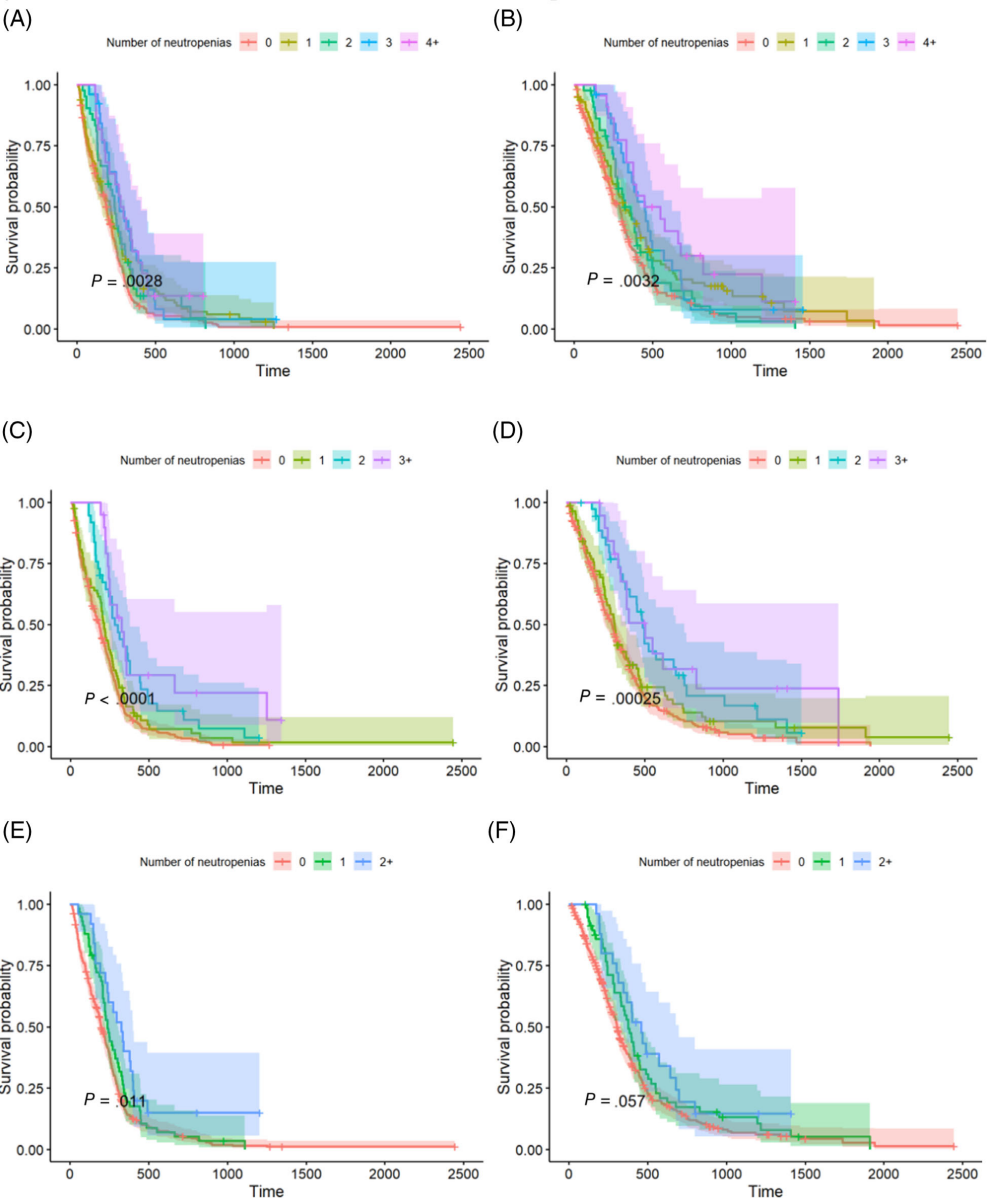
Kaplan‐Meier survival curves of 502 dogs with peripheral nodal B‐cell lymphomas (PNBCL) treated with CHOP (C = cyclophosphamide, H = hydroxydaunorubicin [Doxorubicin], O = Oncovin, P = prednisolone)‐19 and CHOP‐25, stratified by the number of neutropenias associated with vincristine (A, progression‐free survival; B, overall survival), cyclophosphamide (C, progression‐free survival; D, overall survival), and doxorubicin (E, progression‐free survival; F, overall survival).

A Poisson regression model was fitted for each AE variable (grade 1‐5 neutropenic events [NEs] and gastrointestinal toxicity). No significant difference was observed in cyclophosphamide and doxorubicin toxicity between CHOP‐19 and CHOP‐25 protocols, except that cyclophosphamide administration was associated with a lower neutropenia score in CHOP‐25 compared to CHOP‐19. Vincristine administrations were associated with higher maximal gastrointestinal toxicity grade and higher number of NEs in CHOP‐25 compared with CHOP‐19 (Table 4). Gastrointestinal toxicity was not significantly associated with PFS and OS. The number of neutropenia events, highest grade of neutropenia, and neutropenia score all were significantly associated with PFS and OS for each of the 3 chemotherapy drugs, with the number of neutropenia events having the strongest association (Table 4; Figure 1).

In the CHOP‐19 group, 16 (10.3%) dogs were hospitalized once, and 8 (5.2%) dogs twice. In the CHOP‐25 group, 72 (20.7%) dogs were hospitalized once, 8 (2.3%) dogs twice and 2 (0.6%) dogs 3 times. No significant difference in hospitalization rates was noted between CHOP‐19 and CHOP‐25 protocols. Hospitalization was significantly associated with lower PFS and OS (Table 4).

### 3.4 Outcomes

At the time of data collection, 29/502 (5.8%) dogs were still alive, 418/502 (83.3%) were confirmed deceased, and 55/502 (11.0%) were lost to follow‐up, with a similar distribution between CHOP‐19 and CHOP‐25 protocols (*P* = .6). Causes of death were recorded to be definitely caused by lymphoma in 305/418 (73.0%) dogs, whereas it was less clear in 85/418 (20.3%) dogs, and identified as unrelated to lymphoma in 28/418 (6.7%) dogs, with a similar distribution between CHOP‐19 and CHOP‐25 protocols (*P* = .83).

Protocol completion rates between the 2 groups were not significantly different (*P =* .28), with 90/155 (58.1%) dogs treated with CHOP‐19 and 182/347 (52.4%) dogs treated with CHOP‐25 completing the full protocol. The most common reason provided for failure to complete either protocol was PD in 160/230 (69.6%) dogs, followed by toxicity in 20/230 (8.7%) dogs, financial reasons in 9/230 (3.9%) dogs and other reasons in 41/230 dogs (17.8%). Rescue protocols were prescribed in 106/155 (68.4%) dogs induced with CHOP‐19, and in 235/347 (67.7%) dogs induced with CHOP‐25 (*P* = .97). First‐line rescue protocols were CHOP‐based in 33/106 (31.1%) and 59/235 (25.1%) dogs induced with CHOP‐19 and CHOP‐25, respectively (*P* = .3), and lomustine‐based in 42/106 (39.6%) and 110/235 (46.8%) dogs induced with CHOP‐19 and CHOP‐25, respectively (*P* = .26).

The outcome summary of CHOP‐19 and CHOP‐25 protocols is presented in Table 5. Among the 145 dogs with evaluable response when treated with CHOP‐19, 117/145 (80.7%) achieved CR, 24/145 (16.5%) achieved PR, 1 dog maintained SD and 3 dogs developed PD. Among the 337 dogs with evaluable response when treated with CHOP‐25, 276/337 (81.9%) achieved CR, 46/337 (13.6%) achieved PR, 4 dogs maintained SD, and 11 dogs developed PD. No significant difference was found in ORR and CR rates between the 2 protocols (Table 5). As expected, no significant difference was identified in response distribution at the start of the 3rd chemotherapy cycle between CHOP‐19 and CHOP‐25 (*P* = .35), because the 2 protocols differed only after this time point.

**TABLE 5:** Comparison of outcomes between CHOP (C = cyclophosphamide, H = hydroxydaunorubicin [Doxorubicin], O = Oncovin, P = prednisolone)‐25 and CHOP‐19 protocols in dogs with peripheral nodal B‐cell lymphomas (PNBCL).

Variable	CHOP‐19	CHOP‐25	*P*‐value
ORR	141/145 (97.2%, 95% CI, 94.6%‐99.9%)	322/337 (95.5%, 95% CI, 93.3%‐97.8%)	.54
CR	117/145 (80.7%, 95% CI, 74.3%‐87.1%)	276/337 (81.9%, 95% CI, 77.8%‐86%)	.85
6‐month PFS	56.5% (95% CI, 49.2‐65)	56.4% (95% CI, 51.4‐61.9)	.98
1‐year PFS	14.1% (95% CI, 9.4‐21)	17% (95% CI, 13.4‐21.6)	.41
PFS	196 (95% CI, 176‐233)	209 (95% CI, 187‐224)	.22
1‐year OS	36.9% (95% CI, 29.7‐46)	42.8% (95% CI, 37.7‐48.7)	.24
2‐year OS	13.5% (95% CI, 8.6‐21.1)	15.4% (95% CI, 11.7‐20.4)	.61
OS	302 (95% CI, 249‐338)	321 (95% CI, 293‐357)	.61

Abbreviations: CI, confidence interval; CR, complete response; PFS, progression‐free survival; PNBCL, peripheral nodal B‐cell lymphoma; ORR, overall response rate; OS, overall survival.

Dogs in CR at the start of the 2nd chemotherapy cycle achieved longer PFS and OS compared with dogs still in PR (*P* < .001), which was also true for dogs in CR at the start of the 3rd chemotherapy cycle (*P* < .001). Among the dogs with evaluable response, 33/72 (45.8%) dogs still in PR at the start of the 2nd chemotherapy cycle eventually achieved CR, and 10/20 (50%) dogs still in PR at the start of the 3rd chemotherapy cycle eventually achieved CR.

**FIGURE 2:**
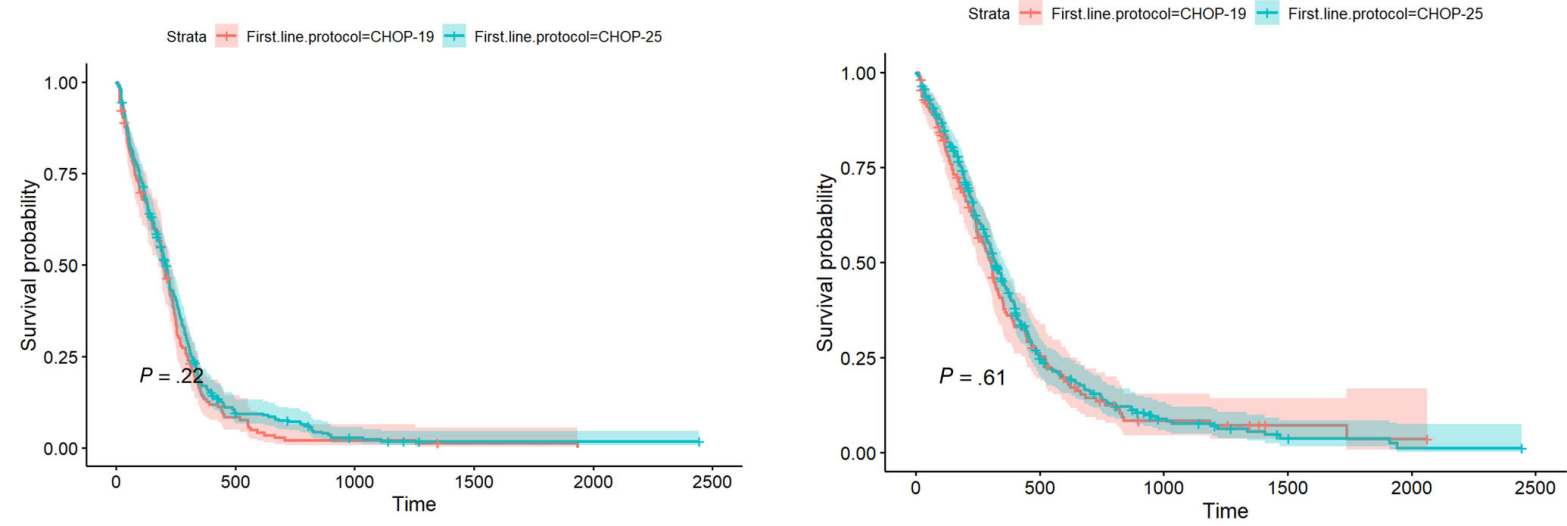
Kaplan‐Meier curves for the progression‐free survival (PFS) (left) and overall survival (OS) (right) in dogs with peripheral nodal B‐cell lymphomas (PNBCL) treated with CHOP (C = cyclophosphamide, H = hydroxydaunorubicin [Doxorubicin], O = Oncovin, P = prednisolone)‐19 (n = 155, red line) and CHOP‐25 (n = 347, green line).

No significant difference in PFS and OS was found between the 2 protocols (Table 5, Figure 2). When adjusting for sex, weight, breed and anemia, and stratifying by substage and median age in the Cox proportional hazards model, there was some suggestion of dogs in the CHOP‐25 group having a lower risk of relapse compared with dogs in the CHOP‐19 group (hazard ratio [HR] = 0.81; 95% CI, 0.66‐1.00; *P* = .05), but this difference was not significant (Table 6). Furthermore, no significant difference in the risk of death between the 2 protocols was identified (HR = 0.89; 95% CI, 0.71‐1.11; *P* = .3) when accounting for sex, weight, breed and anemia, and stratifying by substage and median age.

**TABLE 6:** Summary of fitted model parameters for the cox proportional hazard regression of progression‐free survival (PFS) and overall survival (OS) in dogs with peripheral nodal B‐cell lymphomas (PNBCL) treated with CHOP (C = cyclophosphamide, H = hydroxydaunorubicin [Doxorubicin], O = Oncovin, P = prednisolone)‐25 and CHOP‐19 protocols.

Characteristic	PFS model^a^			OS model^a^		
	HR	95% CI	*P*‐value	HR	95% CI	*P*‐value
Protocol						
CHOP‐19	—	—		—	—	
CHOP‐25	0.81	0.66‐1.00	.05	0.89	0.71‐1.11	.3
Sex						
FE	—	—		—	—	
FN	1.13	0.75‐1.71	.6	1.30	0.84‐2.00	.2
ME	1.60	1.02‐2.50	**.04** *	1.84	1.17‐2.91	**.01** *
MN	1.05	0.69‐1.60	.8	1.21	0.78‐1.87	.4
Weight	1.00	1.00‐1.01	.4	1.01	1.00‐1.02	**.02** *
Breed						
Crossbreed	—	—		—	—	
Border collie	0.94	0.61‐1.45	.8	0.69	0.43‐1.11	.13
Cocker spaniel	1.26	0.73‐2.16	.4	1.15	0.67‐1.98	.6
German shepherd	1.63	0.92‐2.90	.1	1.08	0.59‐1.97	.8
Golden retriever	1.19	0.76‐1.87	.4	1.07	0.67‐1.72	.8
Jack Russell terrier	1.33	0.73‐2.40	.4	1.00	0.48‐2.09	>.9
Labrador retriever	1.08	0.67‐1.73	.8	0.80	0.48‐1.35	.4
Other	1.27	1.00‐1.61	**.05** *	1.07	0.84‐1.37	.6
Anemia	0.99	0.79‐1.25	>.9	1.12	0.88‐1.43	.4

^a^ Stratifying by median age and substage.

* Significant differences (*P* < .05).

Abbreviations: CI, confidence interval; HR, hazard ratio.

No significant difference was found in PFS between the 2 protocols for dogs that were still in PR at the start of the 2nd chemotherapy cycle (*P* = .85), and at the start of the 3rd chemotherapy cycle (*P* = .86), despite a lower chemotherapy DI in CHOP‐25 compared with CHOP‐19.

## 4 DISCUSSION

Our study provided a direct comparison between CHOP‐19 and CHOP‐25 in the 1st‐line treatment of dogs with PNBCL. The cohorts in each group were homogenous, with no significant difference in the baseline characteristics of the dogs. Also, no significant difference was noted in the response distribution between the 2 protocols at the start of the 3rd cycle, also supporting the absence of bias between protocols. Our 1st hypothesis was that CHOP‐19 protocol would be associated with longer PFS because of its higher DI, but such was not the case. No significant difference in 6‐month PFS, 12‐month PFS, median PFS, 1‐year OS, 2‐year OS or median OS was identified between dogs in each group. The ORR, median PFS and OS of dogs in our study are similar to previous reports.^15^, ^16^, ^17^, ^18^ The similar outcomes between the 2 protocols suggest that the 1st 2.5 months are the most important to determine the outcome. As expected, dogs in CR at the start of the 2nd and 3rd chemotherapy cycles had longer PFS and OS compared with dogs still in PR. It was unexpected, however, that dogs still in PR at the start of the 3rd chemotherapy cycle had similar PFS despite CHOP‐25 having its DI decreased by half. Adapting chemotherapy protocols based on the response obtained within the 1st 5 weeks of treatment could improve outcome. This strategy previously was attempted, but the overall PFS and OS remained similar to our current study.^26^ Additional studies are needed to design better protocols.

Despite the CHOP‐25 group having approximately 14% more dogs treated in private practice compared with CHOP‐19, the distribution between academic and private centers for each protocol (CHOP‐19 [40%] and CHOP‐25 [60%]) was similar. The CHOP‐19 group had approximately 12% more dogs treated in UK practices, which also was consistent with approximately 14% more UK centers prescribing CHOP‐19 compared with non‐UK centers. This difference in geographical distribution in the choice of protocol is minimal, and likely reflects a difference in oncologist training. Five of the centers prescribed both CHOP protocols, likely reflecting clinician's or owner's preferences. Advantages of CHOP‐19 over CHOP‐25 are the decreased protocol duration and longer treatment‐free period for the dog, but an advantage of CHOP‐25 is that the financial and logistic burdens for the owners are distributed over a longer time period. No significant difference in PFS or OS was observed in dogs treated in the UK and non‐UK centers, neither at private nor academic institutions. This observation suggests that PNBCL may have similar biological behavior across Europe, and that the prescription of CHOP protocols is consistent with similar effectiveness across referral centers and European countries despite cultural, financial and logistical differences. These findings support the design of future European clinical trials for PNBCL.

Dogs treated with CHOP‐25 had higher numbers of DRs and DDs, most likely associated with vincristine because the higher recorded vincristine gastrointestinal and neutropenia toxicities were the only differences in AEs between the 2 protocols. Because CHOP‐19 maintains a higher chemotherapy DI in the 2nd half of the protocol, we were anticipating the opposite. It was not reported at which point of the protocol the DRs and DDs occurred. Possible hypotheses include: fortnightly administration in CHOP‐25 allows tolerable mild chemotherapy‐induced toxicities and biases centers using CHOP‐25 toward recording more AEs, using higher neutropenia thresholds to implement DRs and DDs, or both.

One of our main findings was confirmation of a strong association between chemotherapy‐induced neutropenia and clinical outcome, as previously suggested.^27^, ^28^ Higher scores, grades, and numbers of episodes of neutropenia all were associated with longer PFS and OS, but the association was stronger with neutropenia. This observation was true with all 3 drugs, but the association was stronger with cyclophosphamide, as approximately 25% of dogs having at least 3 episodes of cyclophosphamide‐induced neutropenia were still in remission at 2 years. One study previously reported that relapses in CHOP protocols occurred most frequently after cyclophosphamide administration and that it should be replaced by another drug.^29^ In a different study, it was indeed shown that the tumor cell decrease rate was lower after cyclophosphamide than with vincristine and doxorubicin.^30^ Nearly 25% of cyclophosphamide responders were still in remission after 1.5 years, however, and the authors suggested that nonresponders were most likely underdosed because the only difference with responders was their significantly higher median body weight of 23.5 kg versus 9 kg. Another study also demonstrated that higher doses of cyclophosphamide are necessary to consistently cause neutropenia in dogs.^31^ In the combination chemotherapy protocol using vincristine, L‐asparaginase, prednisolone, high‐dose cyclophosphamide and doxorubicin (VELPCAP‐HDC), dogs underwent a 12‐week induction followed by a single 500 mg/m^2^ dose of cyclophosphamide, with 12/13 dogs achieving grade 3 or 4 neutropenia. A 3‐year remission rate of 30.8% was obtained.^31^ These studies and ours suggest that optimizing the dosage of cyclophosphamide to obtain consistent neutropenia, and possibly even aiming for grade 3 and 4 neutropenia, may improve the outcome of dogs with PNBCL. Individualized chemotherapy drug escalation recently was attempted but did not seem to improve outcome.^32^ Possible explanations include: weekly chemotherapy administration does not cause clinically relevant myelosuppression, progressive dose escalation may achieve adequate myelosuppression too late in the course of treatment, or the proposed dose escalation and de‐escalation algorithm does not take into account neutrophil count variability. Strategies to achieve more consistent neutropenia from the start of the protocol and spacing chemotherapy administrations at 2‐3 week intervals should be further explored.

Higher number of DRs and DDs were significantly associated with longer PFS and OS, because chemotherapy‐induced neutropenia was the most common cause for dose modification. This finding also is consistent with previous findings.^4^, ^12^ We were not expecting that higher chemotherapy RDI would be associated with shorter PFS and OS, but this finding can be easily explained. Neutropenic events (associated with longer PFS and OS) lead to DRs and DDs, which lead to decreased RDI, demonstrated by the negative correlation between chemotherapy RDI and the number of NEs in our study. Another explanation is the higher RDI associated with CHOP protocols that are discontinued early. The strongest association between higher RDI and shorter outcomes also was obtained for cyclophosphamide, and it was the only drug to maintain a significant association when only completed protocols were considered. In a recent study including 40 dogs with T‐cell lymphomas treated with CHOP, RDI was not significantly associated with PFS and OS, but the study likely was underpowered.^33^ Lower RDI generally has been associated with worse outcomes in humans with diffuse large B‐cell lymphoma treated with R‐CHOP (rituximab + CHOP), although this is not a generalized finding, particularly in the ≥80 years of age subpopulation.^34^ The cause of decreased RDI should be taken into consideration, with planned DRs (eg, in obese patients) likely having a more negative impact on outcome than unplanned DRs (eg, after hematological toxicity).^34^, ^35^, ^36^ Possible explanations for the different impact of RDI in humans may be: CHOP use in humans is designed as a combination protocol and not an alternating protocol, CHOP use in humans is designed with vincristine, cyclophosphamide and doxorubicin administration together, every 2 or 3 weeks, to allow for the occurrence of ≥ grade 2 neutropenias, and higher tolerance of AEs in humans.

Our study had some limitations. First, the number of dogs recruited in the CHOP‐19 cohort was lower than initially intended, thereby decreasing the statistical power. Second, only a minority of European countries were represented, and thus our cohort may not accurately represent the European dog population. Third, the retrospective and multi‐institutional nature of the study meant that some data was incomplete. Fourth, monitoring was not standardized even if the VCOG Response evaluation criteria for peripheral nodal lymphoma in dogs^19^ and VCOG‐CTCAE 1.1^20^ criteria were required for the centers to be able to participate. Fifth, some biases were detected. A significantly higher number of dogs in the CHOP‐25 group received a starting dose of cyclophosphamide lower than the established 250 mg/m^2^ dose, compared with those in the CHOP‐19 group. This initial DR, however, did not negatively impact PFS and OS. More severe vincristine toxicity was recorded in the CHOP‐25 group, which was unexpected and could have been caused by an underlying bias. After thorough analyses, we believe the potential biases in our study are unlikely to have substantially impacted our conclusions. A randomized clinical trial would be necessary to confirm our findings.

We conclude that, in the absence of increased toxicity and similar PFS, CHOP‐19 is a suitable alternative to CHOP‐25. Either protocol could be a reasonable standard of care for future clinical trials. The lack of superiority of 1 protocol over the other, regardless of the response achieved after the 1st half of the protocols, suggests that the 1st 11 weeks have the strongest impact on outcome. Higher number and grade of chemotherapy‐induced neutropenia episodes were strongly associated with higher PFS and OS, in particular for cyclophosphamide. To optimize chemotherapy outcome in dogs with PNBCL, future studies should seek to achieve complete remissions as early as possible, and reach more consistent neutropenia by increasing both the dosages of and intervals between chemotherapy administrations. Our study provides a foundation for the design of future clinical trials in dogs with PNBCL.

## CONFLICT OF INTEREST DECLARATION

Authors declare no conflict of interest.

## OFF‐LABEL ANTIMICROBIAL DECLARATION

Authors declare no off‐label use of antimicrobials.

## INSTITUTIONAL ANIMAL CARE AND USE COMMITTEE (IACUC) OR OTHER APPROVAL DECLARATION

Approved by the Veterinary Ethic Review Committee of the University of Edinburgh before beginning the study (reference 110.21).

## HUMAN ETHICS APPROVAL DECLARATION

Authors declare human ethics approval was not needed for this study.

## Supporting information


**Figure S1:** Boxplots of number of neutropenias against delivered RDI, for vincristine (left), cyclophosphamide (center) and doxorubicin (right). Pearson's correlation coefficient (*r*) and Spearman's rank correlation coefficient (rho) are shown in text on each plot.
