# Supplementary material for: Comparison of CHOP‐19 and CHOP‐25 for treatment of peripheral nodal B‐cell lymphoma in dogs: A European multicenter retrospective cohort study
Source: J Vet Intern Med. 2024 Oct 18;38(6):3193–205. doi: 10.1111/jvim.17222 (PMC11586558; doi:10.1111/jvim.17222)

SUPPLEMENTARY FIGURE 1: Boxplots of number of neutropenias against delivered RDI, for vincristine (left), cyclophosphamide (center) and doxorubicin (right). Pearson's correlation coefficient (r) and Spearman's rank correlation coefficient (rho) are shown in text on each plot.

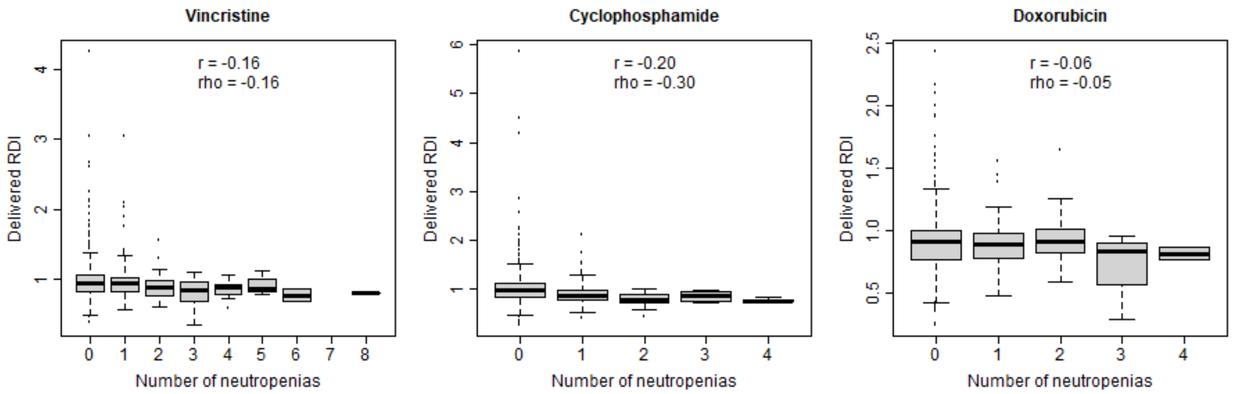

Supplement: Supplementary file 1 — Figure S1: Boxplots of number of neutropenias against delivered RDI, for vincristine (left), cyclophosphamide (center) and doxorubicin (right). Pearson's correlation coefficient (r) and Spearman's rank correlation coefficient (rho) are shown in text on each plot. [file JVIM-38-3193-s001.pdf]
